# Lac Felinum, Provings of

**Published:** 1883-05

**Authors:** S. Swan

**Affiliations:** New York


### PROVINGS OF LAC FELINUM.

S. Swan, M. D., New York.

(1) Mrs. M. B. P., aged about thirty, proved the seventeenth potency.

Great depression of spirits.

Very cross to every one.

Dull pain in forehead, in region of eyebrows.

Heaviness in forehead (clinically verified).

Heavy pressure in sides of head and vertex.

Pulsations in head, with sensation of heat in forehead and constriction across bridge of nose.

Acute pains on vertex.

Acute pains in frontal region.

Intense pain early in morning on vertex and left side of head; commences just in front of vertex with a flush of heat which extends front about an inch, and is followed by the intense pain; the heat and pain then spread (never crossing median line) down left side as a veil, taking half the nose and jaws and entering the ear, causing her to close the eyes from its intensity. During the pain the head was drawn down, so that the chin pressed heavily on chest, and her agony was so great that she had to hold the head firmly in her hands, *and rush through the house from room to room screaming.* (The italicized portion of this symptom has been clinically verified.)

Acute pain over left eye and temple, the agony being so great that she had to hold the head firmly in her hands, *and rush through the house from room to room screaming.*

Pain in head worse from reading.

Sharp lancinating pains passing zigzag down left side of head about every ten minutes, from vertex toward left ear.

Pain commencing with a chilly sensation at root of nose; also a cold pain passing up median line to vertex, and passing down to ear, like the previous symptom.

Pains in all the teeth, as the hot pain from the head touched them.

Intense pain from head along lower jaw, causing mouth to fill with saliva.

Sensation as if tongue were scalded by a hot drink (verified clinically).

Redness under tongue, on gums and whole buccal cavity.

Soreness and sensation of ulcers on tongue and roof of mouth.

The parts of mouth seem to stick together, requiring an injection of air or saliva to separate them.

Loss of taste.

Salivation; tongue enlarged and serrated at edges by teeth.

Brassy taste in mouth.

Small white ulcers covering the tongue and whole buccal cavity.

Elongation of palate.

Tough mucus in pharynx.

Heat in epigastrium.

Slight nausea.

Pain in abdomen and back, as if menstruation was about commencing.

Pain in bowels.

Natural stool, but very slow in passing, at 2 A. M.

Stool long, tenacious, slipping back when ceasing to strain; seeming inability of rectum to expel the contents.

Frequent desire to urinate; urine very pale.

Leucorrhœa ceased on third, and came back on fourth day.

Dryness of rim of glottis.

Very much oppressed for breath—continuing for several days. It is a difficulty in drawing a long breath, or rather that requires the drawing of a long inspiration, for it seems as if the breathing was done by the upper part of lungs alone.

Pain in right side of left wrist when using index-finger.

Dullness, sleepiness, gaping.

Heavy, profound sleep, not easily awakened.

Cold and heat alternately, each continuing but a short time.

(2) The same prover took seven powders of 1m (Fincke), taking one every hour.

Fear of falling down stairs, but without vertigo: fourth, sixth, and seventh days.

Headache on left side, not lasting more than ten minutes at a time: fourth day.

Headache over eyes: sixth and seventh days.

Sharp lancinating pain through centre of left eyeball, leaving it very sore internally, and causing profuse lachrymation: thirtieth day (clinically verified).

Heavy pressure downward of the eyebrows and eyelids, as if the parts were lead: sixth day.

Inclination to keep eyes shut: sixth day.

Eyes feel as if sunken in head, and left eye occasionally waters: first day.

Twitching of outer end of left upper lid, inside: third day.

Cannot bear the smell of clams, of which she is naturally very fond, and cannot eat them: second day.

Stringy tough mucus in pharynx, cannot hawk it up and has to swallow it; when it can be expectorated it is yellow: first day.

Mucus in pharynx between head and throat is thick, yellow, tough, and stringy, expectorated with difficulty, and has a sickish sweet taste: second to seventh days.

Posterior wall of pharynx slightly inflamed, with sensation of soreness: third day.

No appetite: second day (clinically verified).

After eating, feels swollen; has to take off her dress and loosen the clothes: second day.

Great desire to eat paper: second day.

Stomach sore all around just below the belt, worse on left side first day.

Occasionally very slight nausea: second day.

Stomach very sore in epigastric region: second day.

Great sensitiveness of epigastric region: third day.

At midnight, sensation of a cold bandage over lower part of abdomen: third day.

Great weight and bearing-down in pelvis, like falling of the womb, as if she could not walk; worse when standing: first to fifth day.

Pain in pelvis, through hips on pressure, as placing the arms akimbo: third day.

Pain in abdomen as from menses: sixth day.

Furious itching of vulva, inside and out; yellow leucorrhœa: third to sixth day.

Left foot feels cold when touched by right foot: first day.

Legs ache: fifth day.

Dreamed of earthquakes: second day.

(3) Laura Morgan, M. D., gave 200th to a man.

Morbid conscientiousness, every little fault appeared a crime (clinically verified).

Entire right side from crown to sole felt terribly weak, heavy, and distressed, so that it was difficult to walk.

(4) S. Swan, M. D., took a potency, and had a very sore mouth from it.

In addition to what has been published elsewhere by myself, the following symptoms have been cured by me with *Lac Felinum*, and should be compared with the provings:—

Pain in forehead, occiput, and left side of head, with rigidity of cords of neck (splenius and trapezius), and heat in vertex; the pain in forehead is heavy, pressing down over eyes.

Sharp lancinating pain in centre of right eyeball, extending externally to temple and frontal region over eye, with intense photophobia, redness of conjunctiva, and lachrymation; pain worse by reading or writing; the pain appears to be in the interior of eyeball, and extends thence to posterior wall of orbit, and thence to the temples, with throbbing; constipation, loss of appetite, dim sight when reading, lassitude in legs; diagnosed as choroiditis. Cured with 1m and 10m.

Twitching of eyelids, right and left.

Ciliary neuralgia (Dr. Burdick).

Obstruction in urinating, has to wait.

Dragging pain in left ovary.

Constant nervous trembling, especially of hands, as in drunkards with dryness of mouth, and sensation as if tongue were scalded.

Dr. Berridge informs me that he has verified some of the eye symptoms in three remarkable cases. I have had great success with it in eye cases, especially where there is severe pain in back of orbit, indicating choroiditis.

